# Effectiveness of media awareness campaigns on the proportion of vehicles that give space to ambulances on roads: An observational study

**DOI:** 10.12669/pjms.331.12176

**Published:** 2017

**Authors:** Shiraz Shaikh, Lubna A Baig, Maciej Polkowski

**Affiliations:** 1 Dr. Shiraz Shaikh. MBBS, FCPS. Assistant Professor, APPNA Institute of Public Health, Jinnah Sind Medical University, Karachi, Pakistan; 2 Prof. Lubna A Baig. MBBS, FCPS, PhD. Dean, APPNA Institute of Public Health, Jinnah Sind Medical University, Karachi, Pakistan; 3 Maciej Polkowski. Head of “Health Care in Danger” (HCID) Project, International Committee of the Red Cross, Pakistan

**Keywords:** Ambulances, Emergency care, Mass media campaign

## Abstract

**Background and Objective:**

The findings of the Health Care in Danger project in Karachi suggests that there is presence of behavioral negligence among vehicle operators on roads in regards to giving way to ambulances. A mass media campaign was conducted to raise people’s awareness on the importance of giving way to ambulances. The main objective of this study was to determine the effectiveness of the campaign on increasing the proportion of vehicles that give way to ambulances.

**Methods:**

This was a quasi-experimental study that was based on before and after design. Three observation surveys were carried out in different areas of the city in Karachi, Pakistan before, during and after the campaign by trained observers who recorded their findings on a checklist. Each observation was carried out at three different times of the day for at least two days on each road. The relationship of the media campaign with regards to a vehicle giving space to an ambulance was calculated by means of odds ratios and 95% confidence intervals using multivariate logistic regression.

**Results:**

Overall, 245 observations were included in the analysis. Traffic congestion and negligence/resistance, by vehicles operators who were in front of the ambulance, were the two main reasons why ambulances were not given way. Other reasons include: sudden stops by minibuses and in the process causing obstruction, ambulances not rushing through to alert vehicle operators to give way and traffic interruption by VIP movement. After adjustment for site, time of day, type of ambulance and number of cars in front of the ambulance, vehicles during (OR=2.13, 95% CI=1.22-3.71, p=0.007) and after the campaign (OR=1.73, 95% CI=1.02-2.95, p=0.042) were significantly more likely give space to ambulances.

**Conclusion:**

Mass media campaigns can play a significant role in changing the negligent behavior of people, especially when the campaign conveys a humanitarian message such as: giving way to ambulances can save lives.

## INTRODUCTION

Mass media campaigns may be defined as the means by which messages are conveyed through the use of communication channels to a substantial target audience in an effort to modify health beliefs and behaviors.^1^ These communication channels include Television/Cinema advertising, radio broadcasts, use of billboards, magazines, newspapers and transport advertising (signs on buses/taxis). Mass media campaigns have previously been implemented in the field of public health to improve nutrition, promote physical activity, discourage tobacco use, enhance road safety and ameliorate care seeking behaviors in many countries. Campaigns aimed at discouraging cigarette smoking have in the past shown mixed results in their efforts to influence behavior.^2^ Those targeting dietary behavior and physical activity have experienced minor improvements.^3^,^4^ A couple of radio campaigns in African countries on improving health seeking behavior and promoting insecticide treated nets use by pregnant women, have also shown encouraging results.^5^,^6^ However, campaigns aimed at providing road safety education have shown the greatest impact by reducing accidents by up to 9-13%.^7^,^8^

The Healthcare in Danger project was launched in Karachi, Pakistan by the International Committee of the Red Cross in collaboration with APPNA Institute of Public Health in the year 2015. A multicenter research was conducted in Karachi to determine the kinds of violence faced by all cadres of healthcare providers as well as to identify strategies that could prevent and de-escalate violence. One of the main reasons that was identified as the cause of the eruption of violence in health related events was late arrival of ambulances during emergency situations.^9^ The delay was attributed to traffic, people’s negligence on giving way to ambulances and the misuse of ambulance sirens by ambulance drivers.

In light of these results and relevant literature where there is indication of a significantly higher survival of patients who arrived in hospitals earlier during medical emergencies,^10^-^13^ a mass media campaign was conducted in Karachi from march 11^th^ -26^th^ 2016 with the aim of raising people’s awareness on the importance of giving way to ambulances. The campaign involved sending out public awareness messages through billboards, running advertisements and talk shows both on television and radio. Project partners – members of the research team as well as representatives of the ambulance services and other selected interlocutors participated in these programs.

The messaging of the campaign was designed to appeal to the emotions of the population through the use of the slogan “Ambulance ko rasta den, is main aapkaa koi apnaa bhee hosaktaa he (*Give way to the ambulance, it may be carrying someone close to you*)” Parallel to the campaign, three observation surveys were carried out in different areas of the city before, during and after the campaign. These observation surveys were aimed at quantifying the proportion of vehicles that gave way to ambulances on roads and to determine the effectiveness of the campaign as well.

## METHODS

This was a quasi-experimental study with a before and after design conducted in Karachi, Pakistan. Observation surveys were conducted by trained observers who used a checklist designed in the light of findings of HCID Project. Information obtained through the checklist included the time of day, type of ambulance observed, amount of space available to allow changing of lanes, number of vehicles in front of the ambulance and the number of vehicles that created space for the ambulance.

The minimum time that was used as a baseline for not giving space was considered to be fifteen seconds, if space was available. An open ended question was kept to record the main reason as to why the ambulance remained stuck or was blocked in traffic.

Each observation was carried out at three different timings of the day to capture busy traffic as well as low traffic hours in the city of Karachi Pakistan. The timings included Morning hours between 9:00 am - 11:59 am, afternoon hours between 12:00 pm - 4:59 pm and evening hours 5:00 pm - 7:59 pm. The observations were carried out for at least two days on each road. In addition, observations were conducted in six different locations within the city. Ethical approval was obtained from the Institutional Review Board of Jinnah Sind Medical University.

### Statistical Analysis

Data was analyzed using SPSS version 20. Descriptive statistics were represented as frequencies and percentages. The main outcome indicator was based on the percentage of vehicles that gave way to an ambulance when space was available. Percentages of ambulances that were blocked or not given space by at-least one car in front of them and main reasons of not given space were also computed.

The relationship of the media campaign with regards to a vehicle operator giving space to an ambulance was calculated through odds ratios and 95% confidence intervals using multivariate binary logistic regression method.

The relationship was adjusted for time of observation (categorized as rush hours i.e. from 9:00 am to 10:00 am in the morning and from 5:00 pm to 8:00 pm in the evening and non-rush hours), site of observation, type of ambulance (categorized as Suzuki high roof and well equipped) and the number of cars in front of the ambulance (categorized as 1-2, 3-4 and 5 & above) using multivariate binary logistic regression. P-value of <0.05 was considered significant.

## RESULTS

The descriptive characteristics of the observations made are summarized in Table-I. Only those observations were included in which there was at-least one vehicle in front of the ambulance. Overall, 245 observations were included in the analysis. Almost an equal number of observations was made during the three phases i.e. before (n=84), during (n=78) and after (n=83) the media campaign.

**Table-I:** Descriptive Statistics of Ambulances Observed (n=245).

Variable	(n=245)
***Phases***	
Before Media Campaign	34.3%(84)
During Media Campaign	31.8%(78)
After Media Campaign	33.9% (83)
***Timings***	
Rush hours	36.7% (90)
Non-Rush hours	63.3% (155)
***Site of Observation***	
Kalapul	11% (27)
Kalaboard	8.6% (21)
Lasbela Chowk	24.5% (60)
Nazimabad	10.6% (26)
3-Talwaar	26.1% (64)
Numaaish	19.2% (47)
***Type of Ambulance***	
Suzuki High roof	78% (191)
Well Equipped Ambulance	22% (54)
Space to change the lane available	80.4% (197)
Siren Buzing	80.8% (198)
Ambulance rushing through	86.1% (211)
Flashing red light	61.2% (150)
Tailgated by any vehicle	42.4% (104)

More than one third (36.7%) of the observations were made during rush hours. Only 22% of ambulances were properly equipped ambulances while the rest were Suzuki high roofs. In addition, 19.6% of ambulances got stuck in traffic; vehicles in most cases (80.4%) had sufficient space to allow change of lanes.

**Fig. 1:**
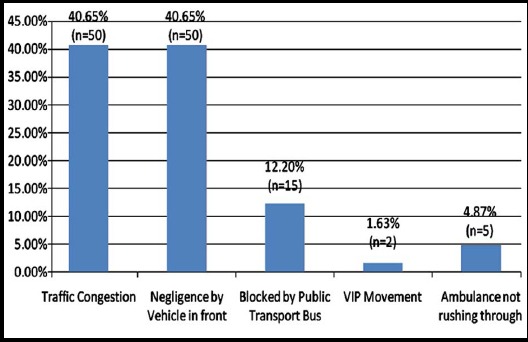
Main Reasons of ambulance not given space (n=122).

In almost a half of the observations (n=122), at-least one vehicle did not give space to an ambulance. Fig.1 shows the main reasons as to why the ambulance was not given space. Traffic congestion and negligence/resistance by the vehicle operator in front of the ambulance were the two main reasons why ambulances were not given space followed by stoppage of minibuses, ambulances not rushing through to seek space and traffic interruptions due to VIP movement.

**Fig. 2:**
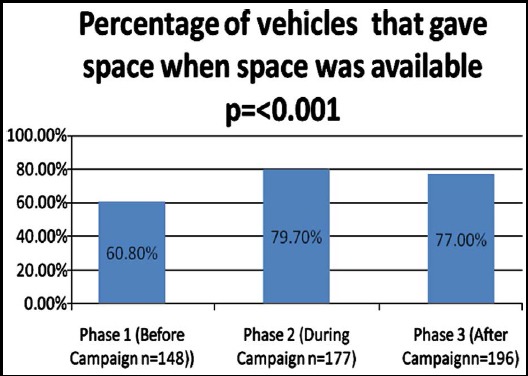
Difference in percentage of vehicles that gave space to ambulance in the three phases.

Among the observations where space to change the lane was available and ambulance was rushing through to seek space (n=172), a total of 521 vehicles in front of the ambulance came under observation. Among all the vehicles, 73.3% responded quickly to ambulances call and immediately gave space. Fig.2 shows the difference in percentages of vehicles that gave space to ambulances in the three phases. Vehicles during (79.7%) and after (77%) the campaign were significantly (p=<0.001) more likely to give space to ambulances as compared to vehicles before the campaign (60.8%).

The unadjusted and adjusted relationship of factors associated with a vehicle giving space to ambulances are shown in Table-II. After adjustments were made on the site, time of day, type of ambulance and the number of cars in front of the ambulance, vehicles during (OR=2.13, 95% CI=1.22-3.71, p=0.007) and after the campaign (OR=1.73, 95% CI=1.02-2.95, p=0.042) were significantly more likely to give space to ambulances.

**Table-II:** Unadjusted and adjusted relationship of factors associated with a vehicle giving space to ambulances (n=521).

Variable	Unadjusted OR (95% CI)	p-value	Adjusted OR (95% CI)	p-value
***Phases***				
Before Media Campaign (n=148)	1.00	<0.001	1.00	0.007
During Media Campaign (n=177)	2.52 (1.54-4.13)	0.001	2.13 (1.22-3.71)	0.042
After Media Campaign (n=196)	2.16 (1.35-3.45)		1.73 (1.02-2.95)	
***Timings***				
Non-Rush hours (n=324)	1.00	0.75	1.00	0.078
Rush hours (n=197)	1.06 (0.71-1.59)		1.50 (0.95-2.35)	
***Site of Observation***				
Kalapul (n=61)	1.00	0.038	1.00	0.110
Kalaboard (n=23)	4.03 (1.07-15.09)	0.025	3.04 (0.77-11.94)	0.063
Lasbela Chowk (n=187)	2.02 (1.09-3.76)	0.055	1.93 (0.96-3.86)	0.207
Nazimabad (n=64)	2.16 (0.98-4.74)	0.245	1.75 (0.73-4.19)	0.757
3-Talwaar (n=107)	1.48 (0.76-2.88)	0.648	0.88 (0.42-1.88)	0.742
Numaish (n=79)	1.30 (0.64-2.63)		0.88 (0.41-1.87)	
***Type of Ambulance***				
Suzuki High roof (n=412)	1.00	0.477	1.00	0.646
Well Equipped Ambulance (n=109)	0.84 (0.52-1.34)		1.13 (0.66-1.91)	
***Number of Vehicles in front***				
1-2 (n=125)	1.00	0.220	0.70 (0.40-1.21)	0.211
3-4 (n=276)	0.71 (0.42-1.21)	<0.001	0.22 (0.11-0.44)	<0.001
5-6 (n=120)	0.31 (0.17-0.56)			

The presence of five or more vehicles in front of an ambulance significantly reduced the chances of an ambulance being given space (OR=0.22, 95% CI=0.11-0.44, p=<0.001). Site of observation, time of day and type of ambulance did not show any significant association after adjustment.

## DISCUSSION

A behavioral improvement of 16-18% from the baseline is a remarkable effect in comparison to the effect of other media campaigns. Although this was the first mass media campaign on the study of ambulances, campaigns aimed at improving transport safety have shown similar behavioral effects ranging from 9-21% difference in comparison to a control group. On the other hand, campaigns targeting cardiovascular disease prevention and physical activity have shown a relatively lesser effect ranging from 1.1%-8.7% difference in comparison to a control group.^14^

The reasons behind the success of the media campaign could have been due to the careful planning in which potential moderators of the campaign’s effectiveness were addressed. First, the campaign was based on the findings of a formative research that singled out barriers associated with the targeted health behavior. Second, the development of core messages was designed by involving all stakeholders including ambulance managers, drivers, healthcare providers and media partners. Moreover, the content of the messages was emotionally motivating and less likely to challenge any traditional or religious behaviors; this enhanced the general acceptability of the message. It is a known fact that behavioral change may be difficult to achieve when messages, that are sent out, face traditional and cultural resistance or require more sustained efforts such as those of quitting smoking or adopting an exercise plan.^15^ This can also be explained by a previous media campaign that was based on television advertisements that were aiming at improving breast feeding practices in Pakistan. The media campaign failed to show any significant improvement.^16^ Third, multiple media sources were used including billboards, television and radio broadcasts. Lastly, the broadcast and display of the commercials and outdoor billboards was paired with engagement from journalists in order to author stories and opinion pieces on the topic. Radio jockeys were put to use in order to include ambulance-related messages into programs that often mention the traffic situations in the city. TV anchors who discuss current affairs and who have a large viewership in Karachi also helped the campaign. This was reflected in the campaign’s marketing survey in which commercials were recalled by 50% of the viewers. Adding to this, the majority of these viewers were able to clearly describe what the message was about. On spot viewership likelihood of billboards was also high. However, a low recall of radio programs was observed, which could be explained by the fact that the medium is mainly used by people in big cities while they drive which makes it difficult for them to grasp the public awareness message.

Although, the campaign has shown significant improvement, literature suggests that in order to achieve a sustained behavioral change over a long period of time, periodic mass media campaigns should be supplemented with other interventions such as law enforcements and direct behavior change communication.^17^,^18^ HCID project is also currently lobbying for enacting legal changes for the right of way of ambulances as well as sensitizing ambulance services to refrain from misusing ambulance sirens.

Apart from negligent behavior from some vehicle operators, other main reasons for the failure to give ambulances way were traffic congestion, obstruction by public transport buses and interruption of traffic due to VIP movement. Therefore, without better traffic management, ambulances will find it difficult to help patients to get timely picked and taken to hospital. Besides the existent laws that urge all vehicle operators to give way to ambulances, there is a need to introduce ambulance friendly traffic rules that allow ambulances to use special passages and that do not bind them to follow protocols of VIP movement.

The strength of this study lies in the fact that assessment is based on spontaneous observation rather than self-reported behavior. Moreover, the relationship of the campaign with regards to giving way to ambulances was adjusted for the different factors including: time of observation, site of observation, type of ambulance and number of vehicles that were present in front of an ambulance. However, the level of education of vehicle operators could not be adjusted since the study was solely based on observation. Another limitation of the study was that it was based on a before and after design which was the only realistic design for the study. Robust evaluation designs such as randomized controlled trials are generally not possible due to the very nature of mass media campaigns,^19^ and it was not possible to find a control point for a mass media campaign that targets the population of a metropolitan city.

## CONCLUSION

Mass media campaigns can play an effective role in changing the negligent behavior of people, especially when the campaigns convey a humanitarian message such as: “*giving way to ambulances can save lives”*. These campaigns should be periodically aired through different media channels for sustainability and should be supplemented with other interventions that address the determinants that are not related to behavior change.
